# Influence of SHH/GLI1 axis on EMT mediated migration and invasion of breast cancer cells

**DOI:** 10.1038/s41598-019-43093-x

**Published:** 2019-04-29

**Authors:** Syeda Kiran Riaz, Yuepeng Ke, Fen Wang, Mahmood Akhtar Kayani, Muhammad Faraz Arshad Malik

**Affiliations:** 1 Department of Biosciences, COMSATS University Islamabad, Islamabad, Pakistan; 2 Centre for Cancer and Stem Cell Biology, Institute of Biosciences and Technology, Texas A&M Health Science Centre, Houston, Texas, USA

**Keywords:** Breast cancer, Morphogen signalling

## Abstract

Sonic Hedgehog signaling is critical for breast morphogenesis and cancer. The present study was conducted to explore the influence of SHH/GLI1 axis on epithelial mesenchymal transition and invasion in breast cancer cells. SHH/GLI1 positive samples demonstrated high expression of Snail and Vimentin with relatively low expression of E-cadherin. Overexpression of Vimentin and Snail in SHH/GLI1 positive patients was also associated with poor overall survival. Interestingly, GANT61 (GLI1 inhibitor) exposure significantly reduced cell viability and induced apoptosis at 10 µM. Suppression of Hedgehog pathway either by CRISPR mediated SHH knock out or GANT61 altered regulation of EMT markers in breast cancer cells. Moreover, in-activation of SHH/GLI1 axis also significantly restricted cell migration and invasiveness. These findings suggest that targeting SHH/GLI1 axis alters expression of EMT markers and abrogates neoplastic invasion in breast cancer cells.

## Introduction

Breast cancer (BC) is affecting a substantially huge proportion of women irrespective of their age worldwide. Metastasis is a frequent event observed in majority of breast cancer patients^1^. Conventional chemotherapy does not offer much hope for these patients while mechanisms underlying tumor spread are still unknown. Epithelial mesenchymal transition (EMT) is a former requisite to initiate dissemination of these cancer cells from the primary site^2^.

Re-activation of developmental pathways in carconigenesis has already been established. In vertebrates, role of Hedgehog pathway as developmental mediator of different organs including axial skeleton patterning has been observed^3^. In mammals, three upstream ligands namely Sonic hedgehog (SHH), Indian hedgehog (IHH) and Desert Hedgehog (DHH) have been identified. Upon activation, these molecules bind with transmembrane receptor known as Patched1 (PTCH1). Binding induced alteration in structural conformation of PTCH1 leads to release of Smoothened (SMO) which mediates downstream activation of GLI family^4^. GLI family consists of three homologous members, GLI1, GLI2 and GLI3 responsible for regulation of Hedgehog targeted genes. SHH exclusively induces the expression of GLI1 while repressing GLI3 with minimal effect on transcriptional regulation of GLI2^5^. GLI1 is mainly regulated at transcriptional level while GLI2 and GLI3 are regulated at protein level as well^6^. Proteolytic cleavage of repressor domain in N-terminus leads to constitutively active GLI2 and GLI3^5^. Furthermore, isoforms of GLI1 specifically truncated GLI1 (tGLI1) have also been shown to promote aggressive phenotypes in human cancers^7–10^. Active GLI1 is considered as a symbol of ligand responsiveness and canonical mode of regulation. Interestingly, it has been reported that SHH and its downstream genes are not activated in GLI1 mutant cells^11^. Moreover, GLI1 mimics SHH in skin and colorectal cancers^12,13^.

Hedgehog signaling mediates EMT during embryonic development as well as cancer metastasis. During mammary morphogenesis, Hedgehog pathway acts as a key regulator in epithelial mesenchymal interactions and tubule maturation^14^. Paracrine signaling of Hedgehog pathway regulates expansion of mammary progenitor cells in intra-epithelial compartment especially during pregnancy^15,16^.

Induction of Hedgehog pathway in mesenchymal cells has been shown to enhance neoplastic transformation^17^. In prostate cancer, tumor growth is influenced by paracrine activation of Hedgehog effectors induced from stromal mass^18^. Also, Hedgehog mediated epithelial stromal interplay enhances invasiveness of breast cancer cells^19^. Moreover, ligand dependent paracrine activation of Hedgehog pathway has been established in several other cancers as well^20^.

SHH mediated activation of GLI1 induces Snail, a major driver of EMT in basal cell carcinoma^21^. Furthermore, GLI1 stimulates Snail, represses E-cadherin and enhances nuclear translocation of β-catenin to induce EMT in skin cancers^22^. SHH-GLI1-Snail axis stimulates EMT in ovarian, pancreatic and neuroendocrine cancers as well^23–25^. However, association of Hedgehog signaling with EMT markers needs further exploration in breast cancer.

GANT61, a small molecule antagonist of GLI1/2 reduces the expression of downstream Hedgehog target genes^26^. Role of GANT61 in curtailing migration and invasion of breast cancer cells has recently been elucidated^27^. Nevertheless, effect of GANT61 on EMT regulation in breast cancer cells still remains elusive.

In the present study, correlation of SHH/GLI1 axis with EMT markers was assessed in breast cancer cohort. Furthermore, impact of SHH knockout using CRISPR/Cas9 and GLI1 inhibition via GANT61 on EMT and cell motility was delineated. Current findings provide firm evidence of involvement of SHH/GLI1 axis in augmenting EMT signals in breast cancer cells.

## Results

### Clinical characteristics of cohort

Breast cancer affected patients included in the present study cohort had a mean age of 45 years ranging from 22 to 80 years. More than half of the cohort (56%) comprised of younger age participants. Majority of patients (84.4%) exhibited moderate to poorly differentiated tumors showing lack of early screening in Pakistan. Most of the patients presented with nodal spread (70.8%) and distant metastasis (12%) indicating higher prevalence of aggressive tumors.

### Over expression of SHH and GLI1 correlates with EMT markers and metastasis in breast cancer patients

**Figure 1:**
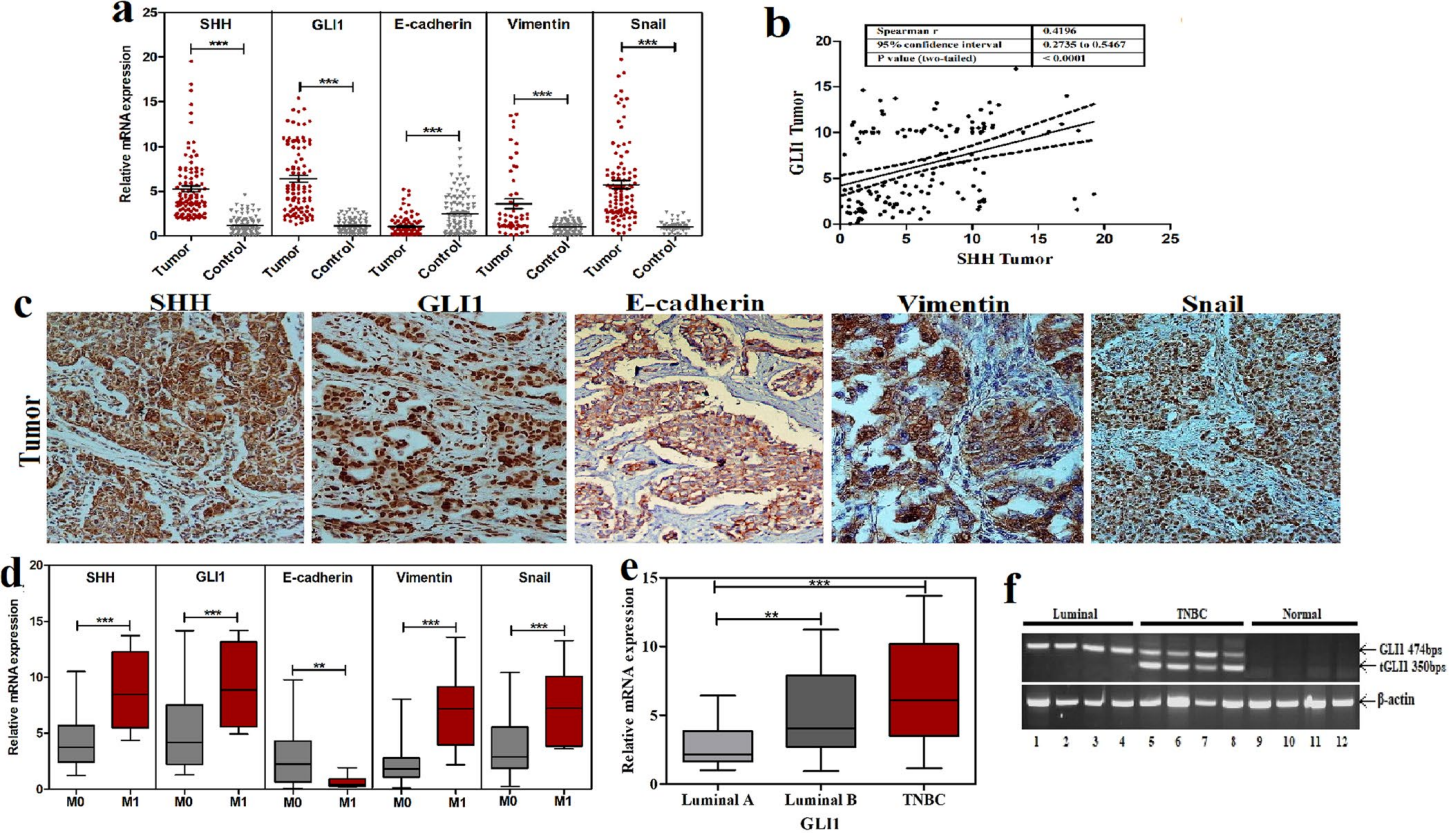
Transcriptional profiling of SHH, GLI1 and EMT markers in breast cancer cohort. (**a**) Scatter plots showing mean relative mRNA expression of SHH, GLI1, E-cadherin, Vimentin and Snail in tumor samples as compared to adjacent normal tissues (Wilcoxon signed rank test, ***p < 0.0001). (**b**) Strong correlation was observed between expression of SHH and GLI1 in tumor samples (Spearman Correlation). (**c**) Immunohistochemical analysis of SHH, GLI1, E-Cadherin, Vimentin and Snail in tumor samples. (**d**) Box plot of SHH, GLI1 and EMT markers showing expressional variation between metastatic and non-metastatic patients (Mann whitney U test, ***p < 0.0001). Expression of GLI1 was significantly higher in luminal B and TNBC subtypes (**e**) while tGLI1 was exclusively found in triple negative patients (**f**). Second quartile of box whiskers plots are median values of data and ends of the whiskers are representative of minimum and maximum data points (**p < 0.01, ***p < 0.0001). Graphical data points and images are representative of at least three independent experiments.

In order to understand association between altered expression of SHH/GLI1 axis and EMT markers in breast cancer cohort, immunostaining and qRT-PCR was performed. Expression of SHH and GLI1 was significantly (p < 0.0001) up-regulated in tumors as compared to respective normal tissues. Substantial proportion of the cohort manifested increase in SHH (92%) and GLI1 (89%) among the patients (Fig. 1a). Strong association was observed between expression of SHH and GLI1 in the tumor samples (r = 0.42, p < 0.0001) (Fig. 1b). GLI2 was also found to be significantly up-regulated in tumor samples but its expression was not associated with SHH (r = 0.002, p = 0.99) (Suppl. Fig. 1a,b). Expression of Vimentin and Snail was markedly up-regulated in the patients with reduced expression of E-cadherin (Fig. 1a). Immunostaining of SHH and GLI1 revealed high expression of these molecules in both epithelial and stromal compartments. Moreover, expression of Vimentin was stromal, E-cadherin was epithelial and Snail was observed in both compartments (Fig. 1c). Demographic data along with clinical assessment of SHH, GLI1, E-cadherin, Vimentin and Snail is enlisted in Table 1. Poorly differentiated tumors significantly overexpressed SHH, GLI1, Vimentin and Snail in comparison to well differentiated tumors. Concordance of expressional alterations of these molecules was observed in advanced stages as well as patients having nodal and distant metastasis (Table 1 and Fig. 1d). Expression of GLI2 was not related to local or distant metastasis in the present cohort (Suppl. Fig. 1a). Based on molecular subtyping, highest expression of SHH was observed in triple negative patients followed by luminal subtypes (p = 0.001). Expression of SHH was not related with HER2 positivity in patients. GLI1 positive patients demonstrated significant increase in luminal B in comparison to luminal A (p < 0.01). Significantly highest transcript levels of GLI1 were observed in TNBC patients (p < 0.0001) (Fig. 1e). In this context, expression of tGLI1 was also analyzed using primers and conditions described previously^8^. Interestingly, tGLI1 was exclusively expressed in triple negative breast cancer patients in comparison to luminal tumors (Fig. 1f). Vimentin and Snail were uniformly expressed in all subtypes while E-cadherin (3.6 ± 2.5) was over-expressed in HER-2 enriched subtype (Table 1).

**Table 1:** Transcriptional profile of SHH, GLI1, E-cadherin, Vimentin and Snail in Pakistani breast cancer cohort and their association with clinical parameters.

Variable	Total	Mean ± SD									
		SHH	p-value	GLI-1	p-value	E-cad	p-value	Vim	p-value	Snail	p-value
Tumor	250	5.2 ± 3.5	<0.0001	6.4 ± 3.8	<0.0001	0.89 ± 1.1	<0.0001	3.6 ± 3.9	<0.0001	5.7 ± 4.4	<0.0001
Control	250	1.2 ± 0.9		1.1 ± 0.8		2.5 ± 2.2		1.0 ± 0.6		1.0 ± 0.4	
**Tumor Size**											
Less than 5 cm	152	5.3 ± 3.5	0.01	5.4 ± 3.7	0.002	2.5 ± 2.2	<0.0001	3.4 ± 3.2	<0.0001	5.7 ± 4.4	0.0007
Greater than 5 cm	98	6.9 ± 4.2		7.2 ± 3.2		0.8 ± 0.8		5.5 ± 3.5		8.6 ± 4.9	
**Grade-wise distribution**											
Grade I	39	4.0 ± 1.7	0.0005	5.9 ± 4.0	0.03	3.7 ± 2.1	<0.0001	1.4 ± 0.7	<0.0001	3.3 ± 1.9	<0.0001
Grade II/ III	211	6.8 ± 4.4		7.3 ± 3.8		0.9 ± 0.9		5.8 ± 3.4		8.7 ± 4.8	
**Stage-wise distribution**											
Stage I/II	152	4.1 ± 1.8	0.002	6.2 ± 4.1	0.04	3.5 ± 2.2	<0.0001	1.5 ± 1.2	<0.0001	3.7 ± 21	<0.0001
Stage III/IV	98	6.6 ± 4.3		7.4 ± 3.5		1.2 ± 1.5		5.5 ± 3.5		8.2 ± 5.0	
**Nodal Involvement**											
N0 (none)	73	3.6 ± 1.6	0.0009	6.5 ± 4.4	0.04	6.5 ± 3.3	<0.0001	1.8 ± 1.6	0.0004	4.4 ± 2.9	0.005
Nodal metastasis	177	6.2 ± 3.8		8.0 ± 3.6		2.0 ± 1.8		4.1 ± 3.4		7.1 ± 4.5	
**Distant Metastasis**											
M0	220	4.3 ± 2.1	<0.0001	5.2 ± 3.5	0.0005	2.7 ± 2.2	0.002	2.3 ± 1.8	<0.0001	3.8 ± 2.3	0.0002
M1	30	8.8 ± 3.4		9.3 ± 3.8		0.6 ± 0.5		7.1 ± 3.3		7.5 ± 3.4	
**Molecular subtypes**											
HER-2	46	2.8 ± 1.3	0.001	2.4 ± 1.6	0.0006	3.6 ± 2.5	0.26	2.7 ± 3.1	0.36	4.5 ± 2.0	0.85
Luminal A	48	4.8 ± 2.8		4.3 ± 2.5		1.9 ± 2.0		3.4 ± 2.5		6.8 ± 5.5	
Luminal B	104	5.4 ± 3.3		6.2 ± 4.1		2.3 ± 2.1		3.3 ± 3.3		5.3 ± 3.9	
Triple Negative	52	6.9 ± 3.7		6.6 ± 3.7		2.6 ± 2.4		3.9 ± 3.6		6.7 ± 5.3	

### Elevated expression of Vimentin and Snail in SHH/GLI1 positive patients is a predictor of poor overall survival

**Figure 2:**
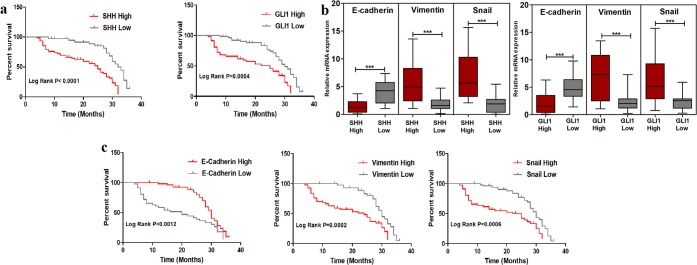
Association of EMT markers with overall survival in SHH/GLI1 positive patients. (**a**) Kaplain Meier plots of SHH and GLI1 showing association with poor overall survival in Pakistani breast cancer cohort. (**b**) Expression profile of E-cadherin, Vimentin and Snail in SHH/GLI1 high and low patients. (**c**) Kaplain Meier plots of E-cadherin, Vimentin and Snail in SHH/GLI1 positive patients showing association with unfavorable outcome. Data is representative of 3 independent experiments (***p < 0.0001).

Association between high expression of SHH, GLI1 and 3-year overall survival (OS) of patients was assessed using Kaplain Meier analysis. Patients expressing high levels of SHH (HR = 3.2, 95% CI = 1.9 to 5.5) and GLI1 (HR = 2.6, 95% CI = 1.5 to 4.5) were found to have unfavorable prognosis (Fig. 2a). High expression of GLI2 was not related with overall survival in the patients (Suppl. Fig. 1c). Majority of patients (85%) were found to be SHH/GLI1 positive and expressional alteration of EMT markers was assessed with Chi squared test and Spearman correlation in patients having high SHH/GLI1 expression. Strong positive association of SHH/GLI1 was observed with Vimentin (r = 0.45, p < 0.05) and Snail (r = 0.56, p < 0.001). On the contrary, reverse correlation of E-cadherin (r = −0.47, p < 0.001) in SHH/GLI1 positive patients (Fig. 2b). Moreover, greater number of SHH/GLI1 positive patients expressed high levels of Snail as well as Vimentin and low levels of E-cadherin (Table 2). These findings discerned potential relationship between SHH/GLI1 axis and EMT markers in breast cancer patients.

**Table 2:** Correlation of Snail, E-cadherin and Vimentin with SHH/GLI1 axis in breast cancer cohort.

Variable	E-cadherin (+ve) N (%)	E-cadherin (−ve) N (%)	χ^2^	p-value
SHH high	30(13)	208(87)	18.9	<0.0001
SHH low	7(58)	5(42)		
GLI1 high	20(8)	225(92)	29.1	<0.0001
GLI1 low	4(80)	1(20)		
	Vimentin (+ve) N (%)	Vimentin (−ve) N (%)		
SHH high	170(71)	68(29)	11.5	<0.001
SHH low	3(25)	9(75)		
GLI1 high	203(83)	42(17)	12.9	<0.001
GLI1 low	1(20)	4(80)		
	Snail (+ve) N (%)	Snail (−ve) N (%)		
SHH high	142(60)	96(40)	8.6	<0.01
SHH low	2(17)	10(83)		
GLI1 high	215(88)	30(12)	9.8	<0.01
GLI1 low	2(40)	3(60)		

High expression of Vimentin (HR = 2.7, 95% CI = 1.6 to 4.7) and Snail (HR = 2.6, 95% CI = 1.5 to 4.6) was predictor of poor prognosis in SHH/GLI1 positive patients. Conversely, high expression of E-cadherin (HR = 0.4, 95% CI = 0.2 to 0.7) was related to favorable outcome in SHH/GLI1 positive samples (Fig. 2c). This data provided evidence of link between over expression of SHH/GLI1 axis and EMT markers in patients having poor survival outcome.

### GANT61 treatment induces cell apoptosis and reduces cell proliferation

**Figure 3:**
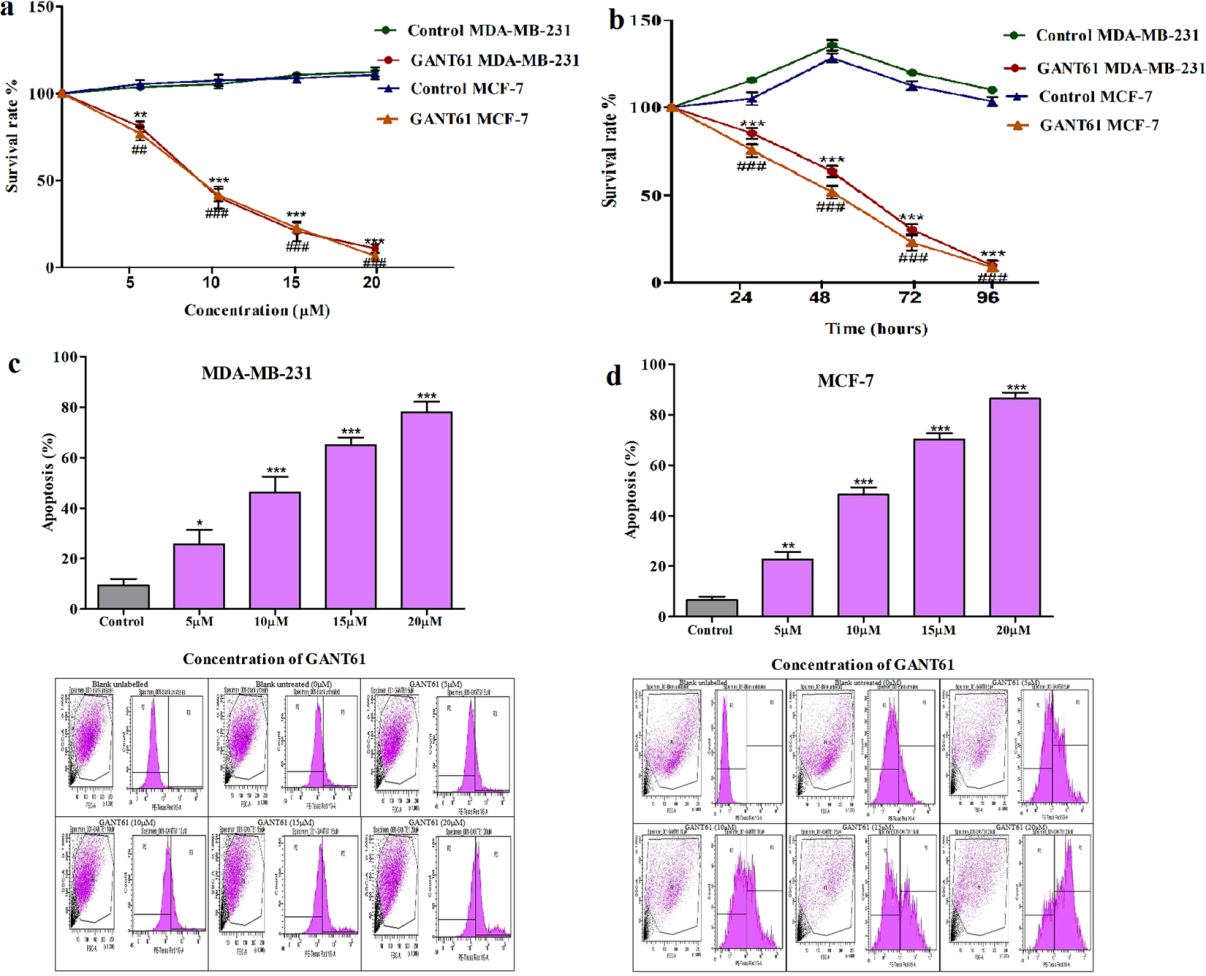
Effect of GANT61 on cell proliferation and apoptosis of breast cancer cells. Cell viability assays were conducted using CCK-8, to observe the effect of GANT61 on cell proliferation in MDA-MB-231 and MCF-7 in both (**a**). Dose dependent (5, 10, 15, 20 µM) and (**b**). Time dependent manner (24, 48, 72, 96hrs). IC50 of GANT61 was determined to be 10 µM. MDA-MB-231 and MCF-7 cells were treated with 10uM GANT61 and the control medium containing DMSO (Unpaired t test, ***, ^###^p < 0.0001). Cell apoptosis was assessed using Annexin V-Cy3 kit and readings were taken after 48hrs of exposure at different concentrations (5, 10, 15, 20 µM) for (**c**). MDA-MB-231 and (**d**). MCF-7. Histograms showing quantitative assessment of apoptosis in c and d. Scatter plots and peaks in first panel of c and d represent untreated, unlabeled control, second panel untreated labeled control, and other panels illustrate different doses of GANT61. All results are representative of three independent experiments.

MDA-MB-231 and MCF-7 cells were selected to examine the effect of Hedgehog inhibition on triple negative and luminal phenotypes. For cell proliferation assays, initially dose-dependence was assessed using different concentrations of GANT61 (0, 5, 10, 15, 20 µM) along with DMSO as control vehicle. GANT61 significantly reduced cell growth by less than 50% at 10 µM in MDA-MB-231 and MCF-7 cells (Fig. 3a). Cells were also exposed to single dose of GANT61 (10 µM) at different time points (0, 24, 48, 72, 96hrs). Significant time dependent growth reduction was observed in MDA-MB-231 and MCF-7 which declined to 63% and 52% respectively (Fig. 3b). For cell apoptosis assays, 50% of the cells became apoptotic in both cell lines at 10 µM concentration after treatment (Fig. 3c,d). Therefore, it was concluded that IC50 of GANT61 was 10 µM and 48hrs were enough to curtail cell growth.

### Suppression of SHH/GLI1 axis inhibits Hedgehog pathway activation in breast cancer cells

**Figure 4:**
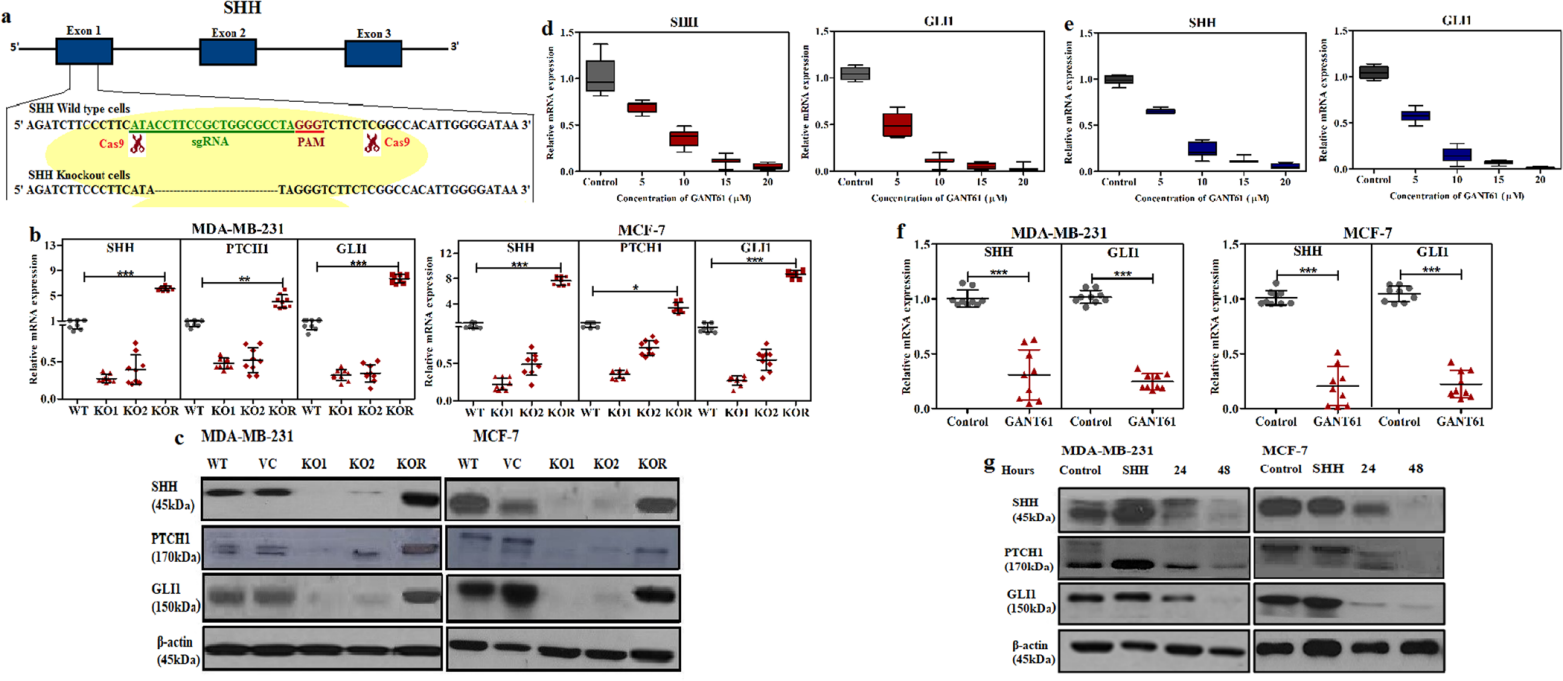
Inhibition of Hedgehog pathway using CRISPR/CAS9 mediated SHH knock-out and GANT61. (**a**). Schematic diagram of CRISPR/CAS9 mediated SHH knockout showing sequence of exon 1 having sgRNA (green), PAM sequence (red) and representative sequence showing modification at cutting site in knockout cells as compared to wild type. Western blot (**b**) and qRT-PCR (**c**) showing effective silencing of SHH, PTCH1 and GLI1 in KO1 and KO2 cells. Expression of SHH, PTCH1 and GLI1 was restored in KO1 rescue (KOR) cells in both lines (Anova with Dunnette post hoc test, *p < 0.05, **p < 0.001, ***p < 0.0001). Box plot demonstrating dose dependent decrease in transcription of SHH and GLI1 after treatment with 10 µM GANT61 in (**d**). MDA-MB-231 and (**e**). MCF-7 cells. Box plots (**f**) and western blots (**g**) showing down regulation of SHH, PTCH1 and GLI1 after 10 µM GANT61 treatment (Unpaired t test, ***p < 0.0001). All results are representative of three independent experiments.

Effect of Hedgehog inhibition on downstream pathway components was observed through CRISPR mediated SHH knockout and GANT61 on SHH/GLI1 axis. MDA-MB-231 and MCF-7 cells were found to express SHH, GLI1 and PTCH1 (Fig. 4). Transgene MDA-MB-231KO1 and MCF-7KO1 demonstrated complete loss of SHH expression. Furthermore, on-target effect of SHH knockout on GLI1 expression was also investigated. GLI1 was significantly down regulated in SHHKO1 of MDA-MB-231 and MCF-7 cells respectively. To further mitigate the concern of off-target effect, 1 µM recombinant SHH (CII24) (R&D Systems, Minneapolis, MN) was added in SHHKO1 cells to rescue ablation of SHH in both knockout cell lines. As SHHKO1 was found to be more effective, drastic recovery of SHH mediated GLI1 expression was observed in MDA-MB-231 and MCF-7 cells (Fig. 4b,c). Effect of GANT61 was also examined to assess its impact on SHH pathway in MDA-MB-231 and MCF-7 cells. Upon GANT61 treatment, expression of GLI1 as well as downstream target gene SHH declined in a dose dependent manner (Fig. 4d,e). GANT61 is reported to inhibit binding of GLI1 to DNA as a result its transcriptional and translational activity was abrogated in both cell lines. mRNA and protein levels of SHH as well as PTCH1 also dwindled in GANT61 treated MDA-MB-231 and MCF-7 cells. Moreover, MDA-MB-231 and MCF-7 wild type cells were also treated with recombinant human SHH (1 μM) to observe induction of GLI1. Expression of PTCH1 and GLI1 increased considerably at mRNA level in SHH treated cells (Fig. 4f,g). This data further strengthened the hypothesis that GANT61 efficiently abrogates the SHH/GLI1 axis in breast cancer cells.

### Inhibition of SHH/GLI1 axis restrains nuclear translocation of GLI1 in breast cancer cells

**Figure 5:**
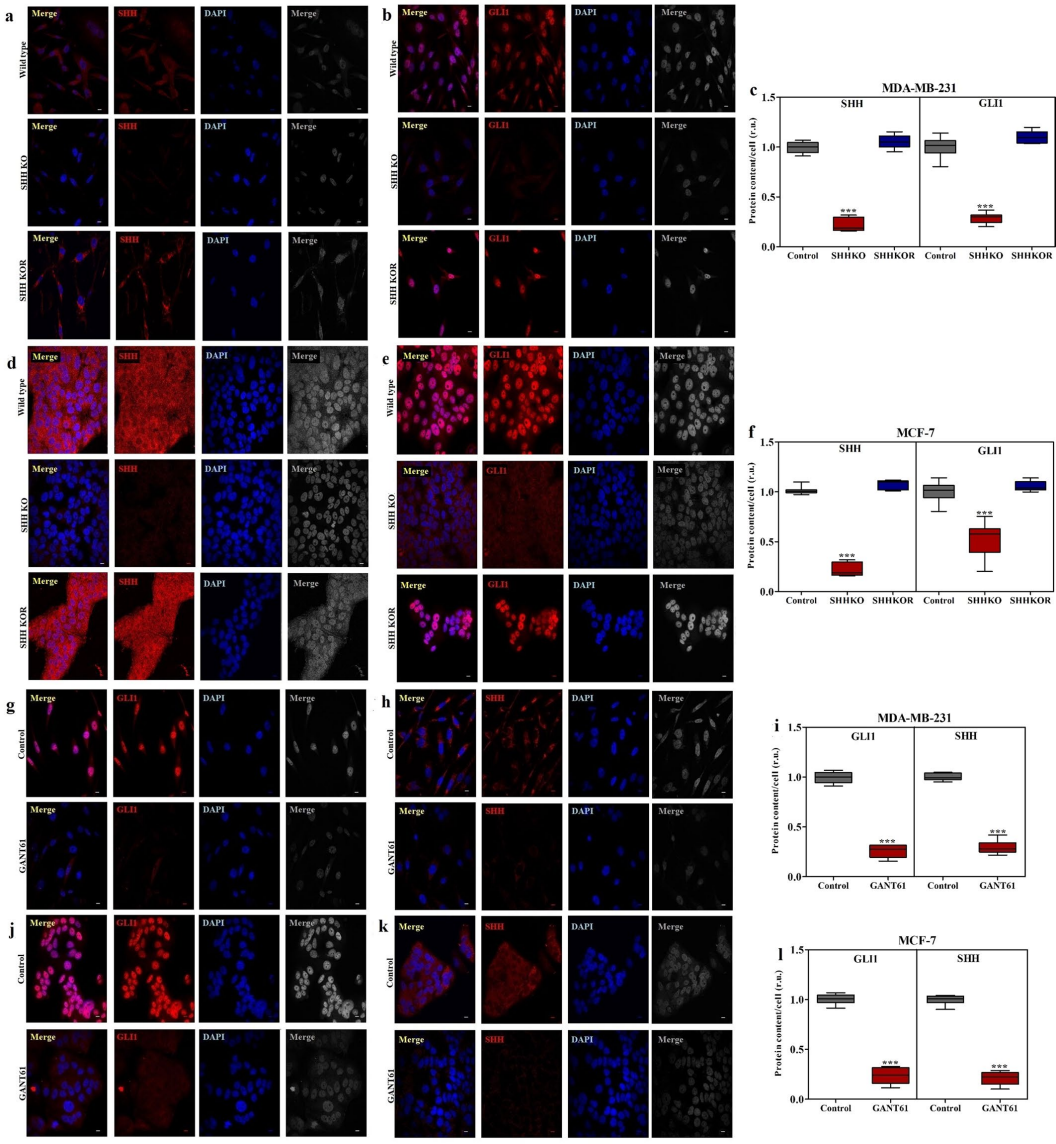
Suppression of Hedgehog pathway activation using CRISPR/CAS9 mediated SHH knock-out and GANT61 in breast cancer cells. Immunofluorescence staining indicating down regulation of SHH and GLI1 in MDA-MB-231 (**a**–**c**) and MCF-7 (**d**–**f**) in CRISPR/CAS9 mediated SHH knockout (SHHKO) and rescued (SHHKOR) cells. Similar decrease in pathway activation was observed after GANT61 (10 μM) treatment in MDA-MB-231 (**g**–**i**) and MCF-7 (**j**–**l**) cells (Scale bar 100 µm). Box plots (**c**,**f**,**i**,**l**) indicate amount of SHH and GLI1 in both cell lines after immunofluorescence quantification in relative units (r.u.). Horizontal lines represent median values and whiskers indicate minimum and maximum values (Anova with Dunnette post hoc test in knockout and Unpaired t test in GANT61 treated cells, ***p < 0.0001). All results are representative of three independent experiments.

Impact of SHH knockout and GANT61 on expression of SHH and nuclear translocation of GLI1 in MDA-MB-231 and MCF-7 cells was evaluated using immuno-fluorescence staining. Nuclear translocation of GLI1 was effectively arrested in SHH knockout MDA-MB-231 and MCF-7 cells. Furthermore, nuclear localization of GLI1 was restored in SHHKOR (rescued) cells in both lines (Fig. 5a–f). Therefore, it was ensued that canonical mode of signaling was responsible for GLI activation in breast cancer cells. Moreover, GANT61 dramatically inhibited translocation of GLI1 into nucleus as was observed in both cell lines after treatment. In addition to that, GANT61 decreased expression of SHH drastically in MDA-MB-231 cells in the cytoplasm as compared with the untreated cells (Fig. 4g–l). These results suggest that SHH/GLI1 axis is potentially active in both ER/PR/HER2 positive and negative cells.

### Halting SHH/GLI1 axis alters expression of E-cadherin, Vimentin and Snail in breast cancer cells

**Figure 6:**
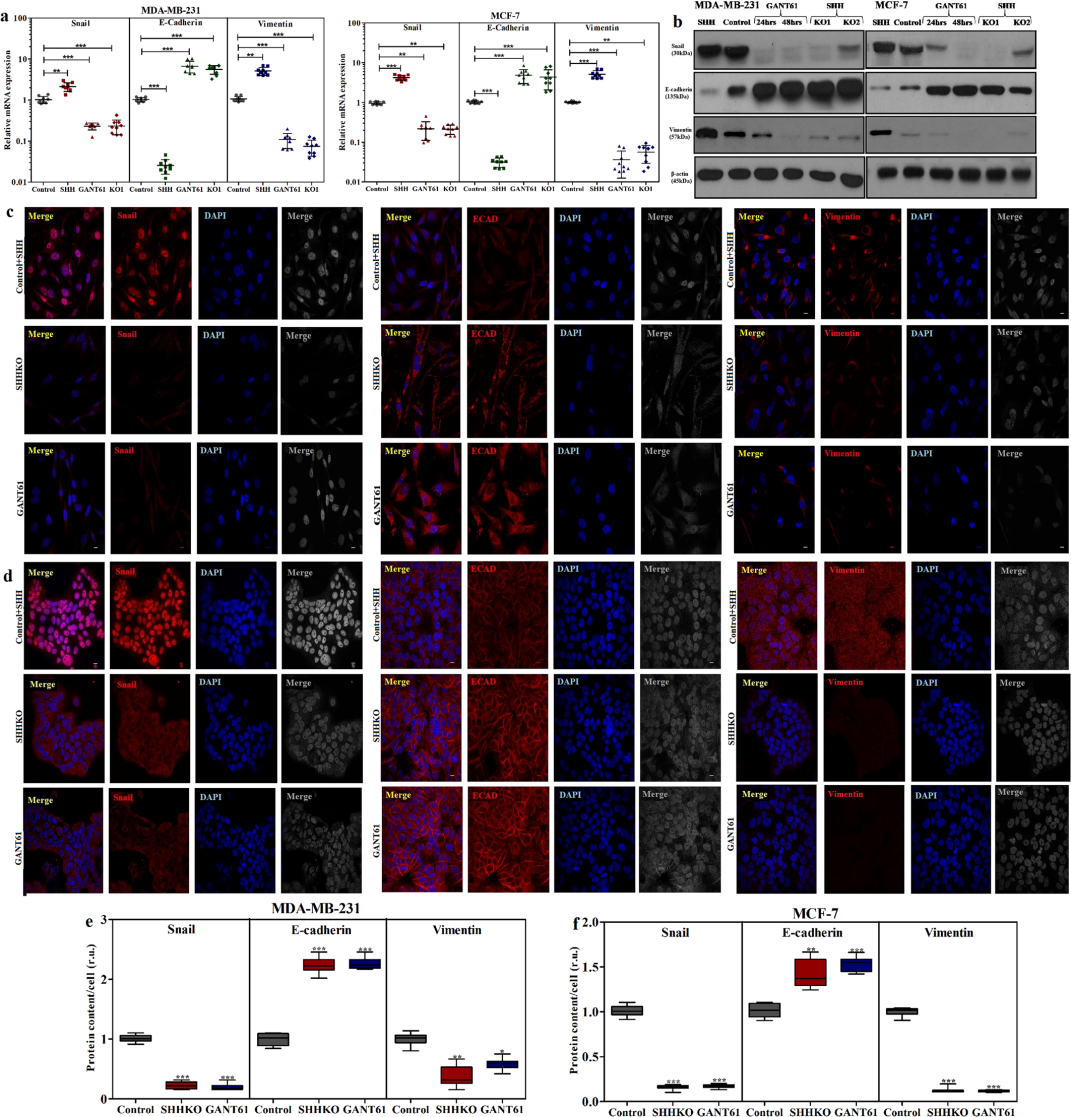
Inhibition of SHH/GLI1 axis alters expression of Snail, E-cadherin and Vimentin in breast cancer cells. Transcriptional variation of EMT markers (Snail, E-cadherin and Vimentin) after CRISPR/CAS9 mediated SHH knockout and GANT61 (10 µM) treatment in (**a**). MDA-MB-231 and MCF-7 cells (**b**). Western blot indicating altered expression of EMT markers after SHH knockout and GANT61 treatment in MDA-MB-231 and MCF-7 cells. Immunostaining showing expression of EMT markers in c). MDA-MB-231 and d). MCF-7 cells in SHHKO and GANT61 (10 µM) treated cells in comparison to wild type cells exposed to recombinant SHH (Scale bar 100 µm). Box plots (**e**,**f**) indicate amount of protein in both cell lines after immunofluorescence quantification in relative units (r.u.). Horizontal lines represent median values and whiskers indicate minimum and maximum values (Anova with Dunnette post hoc test, ***p < 0.0001). All results are representative of three independent experiments.

E-cadherin, Vimentin and Snail are major drivers of metastasis, therefore, affect of SHH knock-out and GANT61 was observed on regulation of these EMT markers. In both cell lines, SHH knock-out and GANT61 (10 μM) treated cells displayed similar expressional alterations at transcriptional and translational levels. Significantly enhanced expression of E-cadherin and decrease in Vimentin and Snail was observed with SHHKO1 cells and GANT61 treatment at mRNA (Fig. 6a) and protein levels (Fig. 6b). Moreover, addition of 1 µM recombinant SHH (CII24) drastically reduced the expression of E-cadherin in MDA-MB-231 and MCF-7 as compared to wild-type control cells. Also, expression of Vimentin was induced in MCF-7 cells upon SHH administration. Furthermore, cellular localization of EMT markers was also assessed in SHHKO1 and GANT61 treated cells using immunofluorescence analysis. Nuclear localization of Snail was stymied in both SHHKO1 and GANT61 treated cells leading to reduction in expression of Vimentin while that of E-cadherin was enhanced (Fig. 6c–f). Therefore, it was confirmed that abrogation of SHH/GLI1 axis have significant impact on regulation of EMT markers in breast cancer cells.

### Abrogation of SHH/GLI1 axis reduces metastatic potential of breast cancer cells by inhibiting motility and invasion

**Figure 7:**
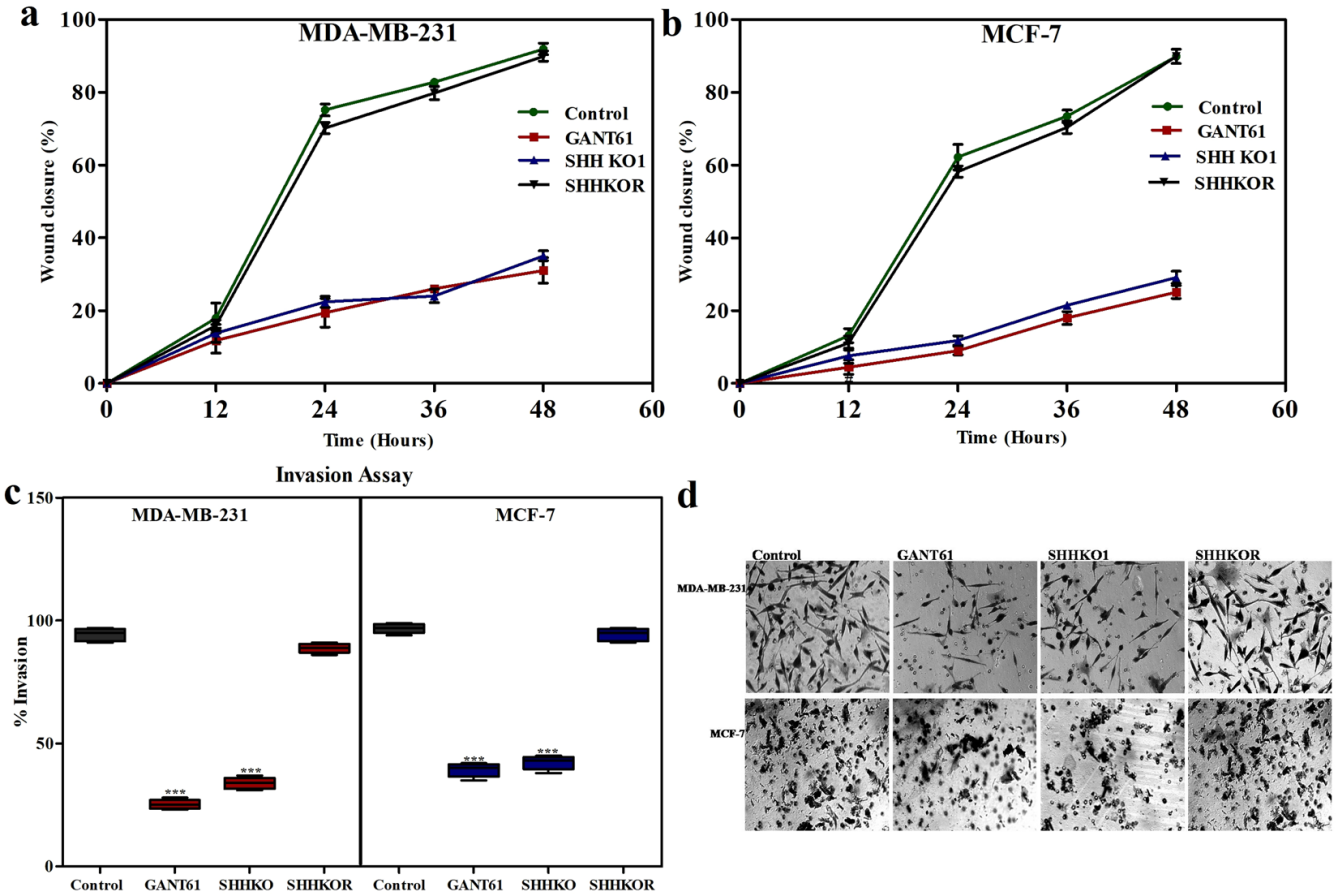
Inhibition of Hedgehog pathway using *in-vitro* models decreases migratory and invasive abilities of breast cancer cells. Wound healing assay was used to assess migration of breast cancer cells following GANT61 treatment, SHH knockout (SHHKO1) and knockout rescue (SHHKOR) in MDA-MB-231 (**a**) and MCF-7 (**b**) recorded after every 12 hours. (**c**) Box plots showing overall difference in invasion of cells after 48hrs measured using transwell assay in both cell lines. Invasion decreased in SHH knockout and GANT61 treated cells while rescued cells showed similar pattern as control cells. Horizontal lines represent median values and whiskers indicate minimum and maximum values (Anova with Dunnette post hoc test, ***p < 0.0001). (**d**) Representative cell invasion picture (Scale bar 50 µm). All results are representative of three independent experiments.

Both SHH knockout cells and GANT61 treated cells exhibited altered expression of EMT markers in MDA-MB-231 and MCF-7 cells. This led us to investigate the influence of SHH knockout and GANT61 on metastatic potential of breast cancer cells. For this purpose, boyden chamber invasion and wound healing assays were used. In MDA-MB-231 cells, wound healing was significantly reduced to 31% in GANT61 treated and 22% in SHHKO cells (Fig. 7a). Similarly, in MCF-7 cells, % wound healing was observed to be 35 and 25 in GANT61 treated and SHHKO cells respectively (Fig. 7b). Likewise, in MDA-MB-231 cells, only 25% cells invaded matrigel after GANT61 treatment and 33% in SHHKO cells. Comparable results were apparent for MCF-7 cells having only 30 and 32% invasion in GANT61 treated and SHHKO cells respectively. SHH knockout rescued (SHHKOR) cells invaded matrigel in a similar manner to wild type cells (Fig. 7c,d). These results further confirmed that SHH/GLI1 inhibition curtails migration and invasion of breast cancer cells.

## Discussion

Aberrant re-activation of Hedgehog pathway has been reported in breast carcinogenesis but influence of SHH/GLI1 axis on EMT and invasion still remains elusive. Strong association was observed between SHH and GLI1 in the patients having aggressive features and poor overall survival as opposed to GLI2. It has been demonstrated that GLI1 does not have a repressor domain and is activated as master regulator of cell proliferation, migration and invasion in several cancers^23,28^. It has also been shown that SHH and its downstream genes are not activated in GLI1 mutant cells^11^. Moreover, GLI1 mimics SHH in skin and colorectal cancers^12,13^. Therefore, SHH mediated GLI1 activation was found to be operational in the present cohort. Also, tGLI1 was found to be exclusively elevated in patients having triple negative breast cancer as opposed to GLI1 which was active in luminal B subtype as well. Transcriptional activation of tGLI1 in TNBC patients have also been observed previously in an American cohort using TMA of 72 patients^10^.

Recently, involvement of SHH-GLI pathway in induction of Snail and repression of E-cadherin has been observed in various cancers^21,23,24^. The present study explored relationship between SHH/GLI1 axis and EMT (Ecadherin, Vimentin and Snail) markers in Pakistani breast cancer cohort. Strong positive correlation of Vimentin and Snail was observed with high SHH/GLI1 expression in the patients. On the contrary, E-cadherin was negatively related to the Hedgehog mediators in the cohort showing the potential involvement of SHH/GLI1 in breast cancer progression. Expression of SHH/GLI1 was found to be negatively correlated with E-cadherin in oral squamous cell carcinoma and pancreatic cancer patients^29,30^. Similarly, reverse correlation was observed between GLI1 and E-cadherin in lung squamous cell carcinoma. Moreover, expression of SHH and GLI1 was found to be high in epithelial cells in contrast to stromal compartment. This might be indicative of tumor mediated paracrine activation of stroma responsible for interplay of markers during epithelial mesenchymal transition.

Impact of SHH/GLI axis inhibition on modulation of EMT and metastasis in breast cancer cells still needs further explication. Furthermore, SMO inhibitors like Vismodegib and Sonidegib have been approved by FDA for treatment of metastatic basal cell carcinoma. Conversely, in breast tumors, trials of these drugs have been terminated in early phases due to futility in metastatic patients^31^. In this regard, GLI inhibitor, GANT61 is paving its way successfully through preclinical evaluations in different cancers including breast^32–35^.

Therefore, effect of GANT61 was evaluated on proliferation and survival of MCF-7 (ER/PR/HER-2 positive) and MDA-MB-231 (ER/PR/HER-2 negative) cells. ER has previously been reported to enhance expression of GLI1 in breast cancer cells^36^. GANT61 (10 µM) was sufficient to reduce growth and induce apoptosis to similar extent in both luminal and triple negative cell lines. Comparable results have been obtained earlier in gastric and pancreatic carcinoma^37,38^.

This is the first study to assess the impact of SHH suppression in breast cancer cells using CRISPR mediated knockout models. In this regard, GANT61 mediated inhibition of GLI1 has been compared with SHH knockout to exploit the avenue of SHH/GLI1 abrogation. Initially, downstream target genes of Hedgehog pathway were examined in SHH knockout, rescued and GANT61 treated cells. It was observed that GANT61 reduced the expression of SHH at both transcriptional and translational levels in a similar manner as SHH knockout eliminated GLI1. Additionally, both SHH knockout and GANT61 inhibited translocation of GLI1 into nucleus providing the evidence for inactivation of GLI1 in breast cancer cells. Sheng *et al*. demonstrated that Thr374 in GLI1 zinc finger domain might be among binding region for nuclear localization signals and is inhibited by PKA to abort translocation of GLI1 into nucleus^39^. Therefore, GANT61 upon binding with GLI1 may not only interfere with DNA binding affinity but also restrict its nuclear translocation. Post translational modifications occurring in GLI1 require further elucidation regarding conformational changes eventuating upon GANT61 binding.

In addition to that, affect of SHH knockout and GANT61 mediated GLI1 inhibition was observed on EMT related biomarkers. GANT61 treated cells responded similarly to SHH CRISPR mediated knockout at 48hrs which was observed to be the peak time for drug effectiveness. Previously, it has been shown that GLI1 modulates EMT by activating Snail which in turn represses E-cadherin and activates Vimentin^22^. It was also observed that exogenous N-SHH significantly reduced E-cadherin in both cell lines, while SHH knockout and GANT61 treatment reversed that effect. Comparable events were observed with Snail and Vimentin in both cell lines having reduction at transcriptional and translational levels after SHH knockout or 48hrs of GANT61 exposure.

This alteration of EMT markers upon SHH/GLI1 inhibition was delineated in *in-vitro* scratch and invasion assays. Invasion and migration of MDA-MB-231 and MCF-7 cells was suppressed by GANT61 and SHH knockout in a similar manner. Moreover, recombinant SHH mediated rescue of Hedgehog ablation reinstated the invasive capability of breast cancer cells. It is thereby inferred that SHH/GLI1 axis enhances invasiveness by potentiating the expression of EMT markers in breast cancer cells.

In conclusion, data suggest that GLI1 reciprocates SHH in breast cancer cells and GANT61 is effectual in curtailing cell viability in dose and time dependent manner. Moreover, GANT61 abrogates the SHH/GLI1 axis to alter EMT switch and deregulates metastatic micro-environment via suppression of migration and invasion. Hence, targeting SHH/GLI1 axis may provide a plausible therapeutic implication for metastatic breast cancer patients.

## Methods

### Clinical characteristics of the cohort

Both ethical and biosafety approvals from COMSATS University (CUI) and Holy Family Hospital (HFH) were taken as per standard guidelines before start of the study. Informed consents from the participants were collected after a thorough briefing of the proposed study. Tumor biopsies (n = 250) along with adjacent normal mammary tissues of breast cancer affected women were collected at the time of surgery. Specimens were collected from September 2013 to December 2014 with a follow-up duration of 36 months till September 2017. Data regarding clinical and pathological findings was obtained in subsequent follow-up from respective laboratory reports and consultation. Tissues were further processed for expression analysis at RNA and protein levels. Methodology was carried out in accordance with the relevant guidelines and regulation.

### Immunohistochemistry

Immunohistochemical staining of SHH, GLI1, E-Cadherin, Vimentin and Snail was performed using tumor sections of 4μm thickness as described previously^27^. Information regarding primary antibodies used in the present study is mentioned in Supplementary Table 2. Immuno-Reactive-Scores (IRS) were evaluated as the product of % of cells positively stained for each molecule categorized from 1 to 4 (1 =< 25%, 2 = 25–50%,3 > 50%). Final IRS scores were ranked as high or low based on mean of IRS.

### Breast cancer cell lines maintenance and culture condition

Breast cancer cell lines (MDA-MB-231 and MCF-7) were generously provided by Dr Yi Li (Breast centre Li, Baylor College of Medicine, USA). These lines were cultured and maintained as per recommendations of ATCC. A prior screening of expression of SHH and GLI1 was performed in both cell lines using qRT-PCR and western blot before generation of knockout models or GANT61 exposure.

### CRISPR mediated knockout of SHH in MDA-MB-231 and MCF-7 cells

CRISPR/Cas9 mediated knockout of SHH molecules was generated in MDA-MB-231 and MCF-7 cells. Briefly, two 20 nucleotide sgRNA targeting SHH were designed using CRISPR design tool (crispr.mit.edu) (Fig. 4a). The sgRNA sequences were flanked on 3′ end with NGG PAM sequence and synthesized by Integrated DNA Technology (IDT). Two sets of sgRNA were used in the study having sequences mentioned in Table 3. LentiCRISPRv2 vector (52961, Addgene) was extracted from gel after being dephosphorylated and digested using BsmB1. Annealed and phosphorylated sgRNA oligos were ligated into the lentiCRISPRv2 vector and transformed into Stbl3 bacterial strains. 293FT cells were cotransfected with SHH lentiCRISPRv2 plasmid, packaging plasmids psPAX2 (12260, Addgene) and pMD2.G (12259, Addgene). Polyethylenimine (23966-2, Polysciences) transfection was used for generation of high titre lentiviruses. After 48hrs, viral supernatant was collected for subsequent infection of MDA-MB-231 and MCF-7 cells. Clonal selection was performed by adding 1 µg/ml puromycin (A11138-02, Gibco) for generation of stable transgenic cell lines. Clones demonstrating disruption of SHH were selected after PCR analysis of exon 1 followed by sequencing and western blot analysis to confirm gene knockout. Cells having SHH lentiCRISPRv2 plasmid were termed as MDA-MB-231KO1, MDA-MB-231KO2, MCF-7KO1 and MCF-7KO2 as compared to wild type MDA-MB-231WT and MCF-7WT cells.

**Table 3:** Sequence of guide RNA for targeting SHH gene.

S.no.	Name	Forward Oligo	Reverse Oligo
1.	KO1	5′CACCG ATACCTTCCGCTGGCGCCTA3′	5′AAAC TAGGCGCCAGCGGAAGGTATC3′
2.	KO2	5′CACCG TAGGGTCTTCTCGGCCACAT3′	5′AAAC ATGTGGCCGAGAAGACCCTAC3′

### GANT61 treatment

GANT61 (G9048) was purchased from Sigma and dissolved in DMSO at a stock concentration of 1 mM solution. DMSO (<0.1%) was used as vehicle control in untreated cells. Briefly, 3 × 10^5^ cells were seeded in 6 well plates until confluency and treated with variable concentrations of GANT61. RNA and protein was extracted from GANT61 treated and untreated wells.

### RNA isolation, cDNA synthesis and Quantitative Real-time PCR

Extraction of total RNA from cell lines and tissue specimens was performed using TRIzol^®^ (15596-018, Invitrogen, USA) as mentioned earlier^40^. cDNA was synthesized using SuperScript^TM^ III Reverse Transcriptase (18080-085, Invitrogen, USA) and random hexamers (48190-011, Invitrogen, USA). Primers were designed and synthesized from IDT and β-actin was used as internal control. Sequences along with their amplicon sizes are provided in the Supplementary Table 1. VeriQuest SYBR Green qPCR Master Mix (75600200RXN, Thermo Scientific, USA) was used for qPCR in *Step One plus* (Applied Biosystem, USA). Reaction conditions included an initial denaturation at 95 °C for 15 min, followed by 40 cycles of denaturation at 95 °C for 15 sec and annealing at 56 °C for 1 min in each cycle. For transcriptional analysis, CT values from qPCR were analyzed using Livak’s method^41^. Data was represented as 2^−∆∆CT^ in terms of fold change ± standard deviation of tumors vs normal mammary tissue.

### Western blot analysis

Total protein content from respective cell lines was collected in radio immune precipitation (RIPA) buffer. Concentration of the harvested proteins was determined using the Pierce BCA protein assay kit (23225, Thermo Scientific, USA). Extracted proteins were separated on 10% SDS-PAGE, transferred to nitrocellulose membrane and blocked by 5% non-fat milk at room temperature. Membranes were incubated with primary antibodies for SHH, PTCH1, GLI1, E-cadherin, Vimentin and Snail (Supplementary Table 2). After overnight incubation at 4 °C membranes were incubated with secondary antibodies at room temperature for 1 hr. Protein signals were visualized using ECL Prime Western Blotting Detection Reagent (GE Healthcare Japan) with β-actin as loading control.

### Immunoflourescence staining

For assessment of protein localization, cells were cultured on gelatin coated cover slips in 24 well. Upon reaching confluency, cells were fixed with 4% paraformaldehyde (PFA), permeabilized with 0.5% Triton X-100 and blocked with 1% bovine serum albumin (BSA). These cells were exposed to primary antibodies (SHH, GLI1, E-cadherin, Vimentin, Snail, Supplementary Table 2 for information on the antibodies) for an overnight incubation at 4 °C. Slides were incubated with Alexa 555-conjugated secondary antibodies (Invitrogen, USA) for 1 hr followed DAPI staining. Images were acquired using Nikon A1si-Spectral Confocal scanning microscope and analyzed using Image J software^42^. Protein content/cell was calculated in relative units (r.u.) as has been described previously^43^.

### Cell viability assay

Briefly, 5 × 10^3^ cells were seeded in 96 well plates, treated with GANT61 and incubated for variable time points. After treatment, CCK-8 (CK04-05, Dojindo, Japan) solution was added to each well and incubated for 4hrs. Absorbance was recorded at 450 nm using BioTek Cytation 3 Cell Imaging Multi-Mode Reader (ThermoScientific, USA) and survival rate was calculated as mentioned earlier^44^. For evaluation of IC50, cells were treated with different doses of GANT61 (0, 5, 10, 15, 20 µM) at different time points (0, 24, 48hrs).

### Apoptosis assay

Briefly, 1 × 10^5^ cells per well were cultured in 6 well plate and treated with variable concentrations of GANT61 (0, 5, 10, 15, 20 µM) for 48hrs. Cells were collected after trypsinization and suspended in 1X binding buffer. Apoptotic cells were then stained by Annexin V-Cy3 (K102-100, BioVision, USA) and analyzed by flowcytometry as per manufacturer’s instructions.

### Wound healing assay

3 × 10^5^ cells were seeded in 6 well plates and after establishment of confluent monolayer, scratch was introduced in the monolayer. Cells were serum starved overnight (16hrs) and mitomycin C (10 μg/ml) was added prior to assay for inhibition of mitosis. Cells were incubated for 2hrs with mitomycin C before the scratch was induced in control and treated or knockout groups. Images of at least 5 different fields were acquired after every 12hrs at different time points (0, 12, 24, 36, 48hrs). Images were analyzed for migration using the Image J software^42^.

### Cell invasion assay

Briefly cylindrical inserts (8μm) pre-coated with 50 μg/ml Matrigel (BD Biosciences, UK) were placed in 24 well plates. Cells (5 × 10^4^) were seeded in the insert having serum free medium and plates were incubated for 24hrs. Inserts were fixed with methanol and stained with crystal violet. Cells were counted under light microscope at 40X magnification as previously described^45^.

### Statistical analysis

Experiments were performed in triplicates and at least twice while data was represented as mean ± S.D. Two-tailed Wilcoxon signed rank test was used to evaluate difference between tumors and adjacent controls (N = 250). Mann Whitney U and Kruskal-Wallis Anova were applied to explore any probable association of molecules with different clinico–pathological parameters (N = 250). Chi-squared test and Spearman correlation was also used to evaluate relationship between all molecules in tumorigenesis. Kaplan-Meier analysis and log rank test was performed for survival analysis (N = 250). Unpaired t-test and Anova with Dunnette post hoc test were applied to assess statistical differences in assays involving cell lines. Statistical analyses were performed using Graphpad Prism 5 (GraphPad Software, Inc. CA, USA). The value for p < 0.05 was considered significant.

## Supplementary information

Influence of SHH/GLI1 axis on EMT mediated migration and invasion of breast cancer cells
