# Supplementary material for: Influence of SHH/GLI1 axis on EMT mediated migration and invasion of breast cancer cells
Source: Sci Rep. 2019 Apr 29;9:6620. doi: 10.1038/s41598-019-43093-x (PMC6488587; doi:10.1038/s41598-019-43093-x)
Supplement: Supplementary file 1 — Influence of SHH/GLI1 axis on EMT mediated migration and invasion of breast cancer cells [file 41598_2019_43093_MOESM1_ESM.docx]

**Influence of SHH/GLI1 axis on EMT mediated migration and invasion of breast cancer cells**

Syeda Kiran Riaz^1,2^, Yuepeng Ke^2^, Fen Wang^2^, Mahmood Akhtar Kayani^1^, Muhammad Faraz Arshad Malik^1*^

^1^Department of Biosciences, COMSATS University Islamabad, Pakistan.

^2^Centre for Cancer and Stem Cell Biology, Institute of Biosciences and Technology, Texas A&M Health Science Centre, USA.

**Corresponding Author**

Dr. Muhammad Faraz Arshad Malik

Department of Biosciences

COMSATS University Islamabad

Pakistan, Zip code: 44000

Phone: +923335524301

Email [famalik@comsats.edu.pk](mailto:famalik@comsats.edu.pk)

**Supplementary table 1: Information of Primers used for qRT-PCR**

| **Primer name** | **Forward primer** | **Reverse primer** | **Product length (bps)** |
| --- | --- | --- | --- |
| **SHH** | CTTCCTCACTTTCCTGGACCG | GGTGGCCGAGTCGTTGT | 136 |
| **GLI1** | CACATCCACAGCCTCTCTTT | CCTGGGTTCTGAAGGAAGATAAT | 110 |
| **tGLI1** | TGTTCAACTCGATGACCC | GTCATGGGGACCACAAGG | 473 |
| **GLI2** | AAGTCACTCAAGGATTCCTGCTCA | GTTTTCCAGGATGGAGCCACTT | 99 |
| **E-cadherin** | CTTCTGCTGATCCTGTCTGATG | TGCTGTGAAGGGAGATGTATTG | 144 |
| **Vimentin** | GGAATTCACTCCCTCTGGTT | CGTGATGCTGAGAAGTTTCG | 105 |
| **Snail** | ACCCCAATCGGAAGCCTAACTACA | AGGACAGAGTCCCAGATGAGCATT | 160 |
| **Ki-67** | GCCTTGGTCTCTTGGGAATAC | GGAGATTAGGAGCCAGTTTGAG | 123 |
| **ER** | CCACCAACCAGTGCACCATT | GGTCTTTTCGTATCCCACCTTTC | 108 |
| **PR** | ATT ACC AGT GTT CCC GTC TTC | CCT GTA CTT CCT CCA GCA TAA | 111 |
| **HER-2** | TTGAGTCCATGCCCAATCC | GTGTTCCATCCTCTGCTGTC | 150 |
| **β-actin** | ATGATATCGCCGCGCTCA | CGCTCGGTGAGGATCTTCA | 150 |

**Supplementary table 2: Antibodies used in the study**

| **Antibody Name** | **Primary Antibody** | **Secondary Antibody** |
| --- | --- | --- |
| **SHH** | (H-160): sc-9024, Santa Cruz Biotechnology, Inc. | Rabbit |
| **GLI-1** | (H-300): sc-20687, Santa Cruz Biotechnology, Inc. | Rabbit |
| **PTCH1** | (H-267): sc-9016, Santa Cruz Biotechnology, Inc. | Rabbit |
| **Vimentin** | (D21H3): XP® mAb #5741, Cell Signaling Technology® | Rabbit |
| **Snail** | (G-7): sc-271977, Santa Cruz Biotechnology, Inc | Mouse |
| **E-cadherin** | (24E10): mAb #3195, Cell Signaling Technology® | Rabbit |
| **β-actin** | (C4): sc-47778, Santa Cruz Biotechnology, Inc | Mouse |


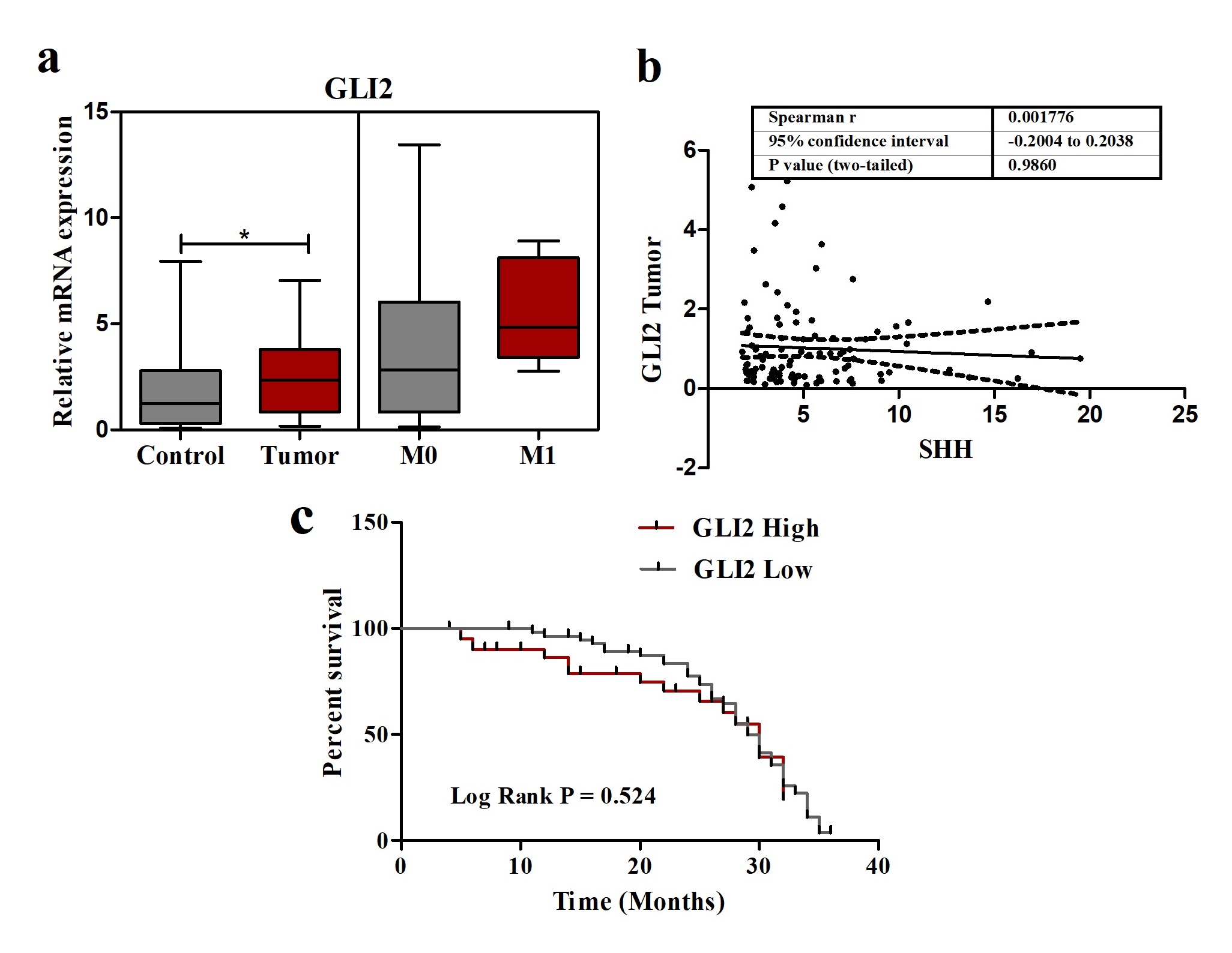


**Supplementary figure 1: Expression analysis of GLI2 in breast cancer cohort and its relationship with SHH.** a). Expression variation of GLI2 among tumor and adjacent controls along with metastatic and non-metastatic samples. b). Expression of GLI2 was not associated with SHH in the present cohort. c). High expression of GLI2 was not related with overall survival of patients in the cohort. (*p<0.05, graphical data points represent mean±S.D. of at least three independent experiments).
